# Supplementary material for: Managing the Mental Health of Healthcare Professionals in Times of Crisis: The Aruban COVID-19 Experience
Source: Healthcare (Basel). 2022 Jul 7;10(7):1263. doi: 10.3390/healthcare10071263 (PMC9318673; doi:10.3390/healthcare10071263)
Supplement: Supplementary file 1 [file healthcare-10-01263-s001.zip › Supplementary File S1.pdf]

Q1. Informed Consent

I agree to participate in this survey.

I acknowledge that should I wish to withdraw from this research at any point I may abandon completing the survey questionnaire and close the screen with no information recorded.

I understand that I will not be asked to identify myself in anyway and that all my responses are completely anonymous.

- ❑ I provide consent and agree to participate.
- ❑ I do not provide consent and do not agree to participate.

Part 1: Participant sociodemographic

Q2. Which GENDER best describes you?

- ❑ Male
- ❑ Female
- ❑ Other

Q3. What is your age? ________________years

Q4. Who do you live with?

- ❑ Alone
- ❑ My partner/spouse only
- ❑ My Children only
- ❑ My partner/spouse and children
- ❑ Parents
- ❑ Friends/flat mates/housemates
- ❑ Other (please specify) ________

Q5. What is your current occupation?

- ❑ Medical staff:
  - ❑ Allied healthcare worker (physiotherapy, ergo-therapist etc.)
  - ❑ Nurse
  - ❑ Physician
  - ❑ Resident
  - ❑ Other
- ❑ Non- medical staff:
  - ❑ Administrative
  - ❑ Manager
  - ❑ Supportive (e.g. maintenance, restaurant, housekeeping)
  - ❑ Technician
  - ❑ Other ___________________

Q6. How many years have you been employed?

Years employed	< 1 year	1-5 years	6-10 years	> 10 years
In the health care sector	❑	❑	❑	❑
In Dr. Horacio E. Oduber Hospital	❑	❑	❑	❑

Q7. COVID-19 is known to be more dangerous for people with certain risk factors. Please check the box with the risk factor that best applies to you. ***(you can choose more than 1 option)***

- ❑ None
- ❑ Lung disease
- ❑ Diabetes
- ❑ Chronic kidney disease
- ❑ Heart disease
- ❑ Smoking
- ❑ Liver disease
- ❑ Obesity
- ❑ Previous psychiatric illness
- ❑ Immunocompromised (Cancer, HIV or taking immune weakening medication)
- ❑ Currently pregnant
- ❑ Other sickness (please specify) ____________________

Q8. In your perception, how would you rate your PERSONAL risk of contracting COVID-19 in the workplace during COIVD-19 outbreak?

- ❑ Low risk
- ❑ Moderate risk
- ❑ High risk

Q9. Thinking about your answer to question 8, which of the following factors makes you feel concerned and how serious would you rate it?
0=not at all; 1=slight; 2=moderate; 3=very much

	0	1	2	3
My profession as a healthcare worker	❑	❑	❑	❑
My working department or office	❑	❑	❑	❑
My age	❑	❑	❑	❑
My other underlying conditions	❑	❑	❑	❑
My long working hours	❑	❑	❑	❑

Part 2: Concerns/worries

Q10. What is your level of concern for each of the following regarding COVID-19?
0=not at all; 1=slight; 2=moderate; 3=very much

	0	1	2	3
Fear for getting infected	❑	❑	❑	❑
Fear of losing financial stability or job	❑	❑	❑	❑
Initial uncertainty about mode of transmission, infectiousness and transmissibility	❑	❑	❑	❑
Risk of passing the infection to my family members	❑	❑	❑	❑
Managing family responsibilities during the lock-down (e.g. child day care, home schooling)	❑	❑	❑	❑

Q11. What is your level of concern regarding each of the following?
0=not at all; 1=slight; 2=moderate; 3=very much

	0	1	2	3
Availability of Personal Protective Equipment for Health Care Worker's	❑	❑	❑	❑
Caring for COVID-19 patients or persons under investigation for COVID-19	❑	❑	❑	❑
Availability of testing kit and the duration of test result	❑	❑	❑	❑
Number of staff available to take care of COVID-19 patients or patients under investigation	❑	❑	❑	❑
Availability of hospital beds/isolation units needed	❑	❑	❑	❑
Adequate supply of cleaning and sanitation material	❑	❑	❑	❑
Fast changing protocols/guidelines for managing patients	❑	❑	❑	❑
Level of training of Health Care Worker's about COVID-19	❑	❑	❑	❑
Response of the public to the lockdown	❑	❑	❑	❑

If there are any concern not listed in the questions above, please notify in this box ?

Q12. What are your concerns as a healthcare worker if you have, or should test positive for COVID-19?
0=not at all; 1=slight; 2=moderate; 3=very much

	0	1	2	3
I have no self-quarantine space at home	❑	❑	❑	❑
I am worried that I may infect family members unintentionally	❑	❑	❑	❑
I am worried about being isolated without contact with family members	❑	❑	❑	❑
I am worried of being stigmatized after being tested positive	❑	❑	❑	❑

I am worried that I can die due to lack of specific treatment	❑	❑	❑	❑
I am worried that I can lose my job	❑	❑	❑	❑

Q13. Have you treated/provided care for a patient diagnosed with COVID-19?

- ❑ Yes
- ❑ No

Part 3: Organization

Q14. Have you received any of the following instruction/training of COVID-19?

	Yes	No
Screening people for COVID-19	❑	❑
Treatment guidelines	❑	❑
Guidelines on management of suspected cases on non-COVID-19 wards	❑	❑
The test that should be performed to make the diagnosis	❑	❑
Training on donning (putting on) and doffing (taking off) of Personal Protective Equipment	❑	❑
Training on hand hygiene	❑	❑
Patient transportation	❑	❑
Visitor policies	❑	❑

Q15. Did you need additional training about Covid-19?

- ❑ Yes
- ❑ No

Q16. Are you satisfied with the quality of information you received from your workplace about COVID-19?

___

- ❑ Yes
- ❑ No (please specify why) ____________________

___

Q17. Do you think that all staff in your unit adequately followed infection control policies, rules and guidelines?

___

- ❑ Yes
- ❑ No

___

Q18. In my opinion, my workplace is well equipped to deal with COVID-19?
0=not at all; 1=slight; 2=moderate; 3=very much

	0	1	2	3
The preparedness of my workplace when it comes to organization is adequate	❑	❑	❑	❑
The preparedness to stand by me and my colleagues when it comes to psychological safety is adequate.	❑	❑	❑	❑
The preparedness of my workplace with respect to required equipment is sufficient.	❑	❑	❑	❑

Q19. Do you think that your hospital is prepared for future infection outbreaks (similar to COVID-19) in general?

___

- ❑ Yes
- ❑ No
- ❑ I don't know

___

Part 4: General wellbeing

Q20. The following questions relate to your personal subjective wellbeing by using (Personal Wellbeing Index-scale). The following questions ask about how satisfied you feel at this moment.

0 = no satisfaction at all, 10= completely satisfied.

Thinking about your own life and personal circumstances:

1. How satisfied are you with your **standard of living**?

   0 ☐ 1 ☐ 2 ☐ 3 ☐ 4 ☐ 5 ☐ 6 ☐ 7 ☐ 8 ☐ 9 ☐ 10 ☐

2. How satisfied are you **with your health**?

   0 ☐ 1 ☐ 2 ☐ 3 ☐ 4 ☐ 5 ☐ 6 ☐ 7 ☐ 8 ☐ 9 ☐ 10 ☐

3. How satisfied are you with **what you are achieving in life**?

   0 ☐ 1 ☐ 2 ☐ 3 ☐ 4 ☐ 5 ☐ 6 ☐ 7 ☐ 8 ☐ 9 ☐ 10 ☐

4. How satisfied are you **with your personal relationship**?

   0 ☐ 1 ☐ 2 ☐ 3 ☐ 4 ☐ 5 ☐ 6 ☐ 7 ☐ 8 ☐ 9 ☐ 10 ☐

5. How satisfied are you **with how safe you feel**?

   0 ☐ 1 ☐ 2 ☐ 3 ☐ 4 ☐ 5 ☐ 6 ☐ 7 ☐ 8 ☐ 9 ☐ 10 ☐

6. How satisfied are you **with feeling part of your community**?

   0 ☐ 1 ☐ 2 ☐ 3 ☐ 4 ☐ 5 ☐ 6 ☐ 7 ☐ 8 ☐ 9 ☐ 10 ☐

7. How satisfied are you with **your future security**?

   0 ☐ 1 ☐ 2 ☐ 3 ☐ 4 ☐ 5 ☐ 6 ☐ 7 ☐ 8 ☐ 9 ☐ 10 ☐

Q21. The following questions relate to your psychological distress level. Please indicate how often the following has occurred in your life at home and at work in the past 4 weeks thinking about the COVID-19

1 = none of the time, 2 = a little of time, 3= some of the time, 4= most of the time, 5 = all the time

	1	2	3	4	5
1. How often did you feel tired out of no good reason?	❑	❑	❑	❑	❑
2. How often did you feel nervous?	❑	❑	❑	❑	❑
3. How often did you feel so nervous that nothing could calm you down?	❑	❑	❑	❑	❑
4. How often did you feel hopeless?	❑	❑	❑	❑	❑
5. How often did you feel restless or fidgety?	❑	❑	❑	❑	❑
6. How often did you feel so restless you could not sit still?	❑	❑	❑	❑	❑
7. How often did you feel depressed?	❑	❑	❑	❑	❑
8. How often did you feel that everything was an effort?	❑	❑	❑	❑	❑
9. How often did you feel so sad that nothing could cheer you up?	❑	❑	❑	❑	❑
10. How often did you feel worthless?	❑	❑	❑	❑	❑

Q22. Have you used any coping strategies to alleviate stress during COVID-19?

- ❑ Yes
- ❑ No

---

*If you choose **No** please continue with Q23.*

Q23. If you choose Yes, which personal coping strategies have you used to alleviate stress during COVID-19?

0=never; 1=sometimes; 2=often; 3=always

	0	1	2	3
1. Followed strict personal protective measures (e.g., mask, gown, hand washing etc.)	❑	❑	❑	❑
2. Read about COVID-19 and how to prevent it	❑	❑	❑	❑
3. I avoid being in the hospital unnecessarily to avoid exposure	❑	❑	❑	❑
4. I Avoid going out in public places to minimize exposure	❑	❑	❑	❑
5. Did relaxation activities, e.g., involved in meditation/prayers, sports, exercise etc.	❑	❑	❑	❑
6. Avoided media news about COVID-19 and related fatalities	❑	❑	❑	❑
7. I received professional help from a professional	❑	❑	❑	❑

Preferred support

The Peer Support network is a task force implemented with the goal to offer psychological support to health care workers. There are three main levels of support:

1. *Individual level support:* access to self-help resources directly online or paper format
2. *Peer level support:* support provided by peers to individuals or groups
3. *Expert level support:* provision of professional help e.g. Psychiatrist, psychologist, social worker etc.

Q24. What kind of support would you need from your workplace?

- ❑ Individual level support
- ❑ Peer level support
- ❑ Expert level support
- ❑ No psychological support needed
- ❑ I don't know

Q25. Are there wellbeing support services available to you through your workplace?

____

- ❑ Yes
- ❑ No
- ❑ Don't know

____

Q26. What is your single greatest work-related need at this moment?

Thank you for participating in the Needs Assessment Survey for Health Care workers of HOH.

Your response is incredibly valuable, and we appreciate the time you have invested.
The outcome will be reported to the staff of the dr. Horacio E. Oduber Hospital.

References:
Q21. Personal wellbeing index- ENG. http://www.acqol.com.au/uploads/pwi-a/pwi-a-english.pdf
Q22. Kessler et al. https://www.tac.vic.gov.au/files-to-move/media/upload/k10_english.pdf
Q23. Imran Khalid et al. Healthcare Workers Emotions, Perceived Stressors and Coping Strategies during a MERS-CoV Outbreak. Clinical Medicine & Research. March 1, 2016 vol. 14 no. 1 7-1
