# Supplementary material for: Managing the Mental Health of Healthcare Professionals in Times of Crisis: The Aruban COVID-19 Experience
Source: Healthcare (Basel). 2022 Jul 7;10(7):1263. doi: 10.3390/healthcare10071263 (PMC9318673; doi:10.3390/healthcare10071263)
Supplement: Supplementary file 1 [file healthcare-10-01263-s001.zip › Supplementary File S2.pdf]

Supplementary File S2: Responses to the questionnaire analysed by the profession.

Table 1. Staff feelings during 1st wave of COVID-19 outbreak among different position (Fig 2 and 3)

Questions	Condition	Nurses N = 117	Doctors N= 34	Other staff N = 80	x^2	*p* -value
1. The rate of your personal risk of contracting COVID-19 in the workplace during the outbreak[a]	Low risk	32 (27)	10 (35)	37 (46)		
	Moderate risk	45 (39)	10 (35)	31 (39)		
	High risk	40 (34)	9 (31)	12 (15)		
	**Mean ± SD**	**2.07 ± 0.79**	**1.97 ± 0.82**	**1.69 ± 0.72**	**12.64**	**0.013**
2. Your profession as a healthcare worker makes you feel concerned[b]	Not at all	9 (8)	3 (9)	27 (34)		
	Slight	22 (19)	7 (21)	30 (38)		
	Moderate	41 (35)	20 (59)	15 (19)		
	Very much	45 (39)	4 (12)	8 (10)		
	**Mean ± SD**	**2.04 ± 0.94**	**1.74 ± 0.79**	**1.05 ± 0.97**	**57.80**	**< 0.001**
3. Your department or office makes you feel concerned[c]	Not at all	9 (8)	6 (18)	24 (30)		
	Slight	29 (25)	13 (38)	26 (33)		
	Moderate	51 (44)	8 (24)	22 (28)		
	Very much	28 (24)	7 (21)	8 (10)		
	**Mean ± SD**	**1.84 ± 0.88**	**1.47 ± 1.02**	**1.18 ± 0.98**	**26.09**	**< 0.001**
4. Fear for getting infected[b]	Not at all	7 (7)	7 (21)	16 (22)		
	Slight	30 (28)	14 (42)	31 (43)		
	Moderate	45 (42)	7 (21)	17 (23)		
	Very much	26 (24)	5 (15)	9 (12)		
	**Mean ± SD**	**1.83 ± 0.87**	**1.30 ± 0.98**	**1.26 ± 0.94**	**21.64**	**0.001**
5. Fear of losing financial stability or job[b]	Not at all	7 (7)	7 (21)	12 (16)		
	Slight	21 (20)	9 (27)	19 (26)		
	Moderate	36 (34)	10 (30)	14 (19)		
	Very much	43 (40)	7 (21)	28 (38)		
	**Mean ± SD**	**2.07 ± 0.92**	**1.52 ± 1.06**	**1.79 ± 1.1**	**13.02**	**0.043**
6.You were uncertain about the mode of transmission, infectiousness and transmissibility of SARS-CoV-2[a]	Not at all	9 (8)	11 (33)	22 (30)		
	Slight	22 (20)	13 (39)	22 (30)		
	Moderate	52 (48)	8 (24)	20 (27)		
	Very much	25 (23)	1 (3)	9 (12)		
	**Mean ± SD**	**1.86 ± 0.87**	**0.97 ± 0.85**	**1.22 ± 1.01**	**32.40**	**< 0.001**
7.You were concerned of passing the infection to your family members[a]	Not at all	10 (9)	3 (9)	8 (11)		
	Slight	13 (12)	7 (21)	10 (14)		
	Moderate	24 (22)	10 (30)	26 (36)		
	Very much	61 (56)	13 (39)	29 (40)		
	**Mean ± SD**	**2.26 ± 0.99**	**0.97 ± 0.85**	**2.04 +/_ 0.99**	**7.69**	**0.261**
8.You were concerned about the availability of Personal Protective Equipment for HCW[c]	Not at all	9(8)	2 (6)	16 (22)		
	Slight	21 (19)	12 (36)	23 (32)		
	Moderate	32 (30)	16 (49)	21 (29)		
	Very much	46 (43)	3 (9)	13 (18)		
	**Mean ± SD**	**2.06 ± 0.98**	**1.61 ± 0.74**	**1.42 ± 1.0**	**29.43**	**< 0.001**
9. Youre were concerned about caring for COVID-19 patients or persons under investigation for COVID-19[c]	Not at all	9 (8)	5 (15)	24 (33)		
	Slight	32 (30)	13 (39)	22 (30)		
	Moderate	31 (29)	10 (30)	18 (23)		
	Very much	36 (31)	5 (15)	9 (11)		
	**Mean ± SD**	**1.87 ± 0.98**	**1.45 ± 0.94**	**1.16 ± 1.03**	**25.46**	**< 0.001**
10.You were concerned about the availability of testing kit and the duration of test result[c]	Not at all	12 (11)	10 (31)	24 (33)		
	Slight	32 (30)	11 (34)	22 (30)		
	Moderate	33 (31)	9 (28)	18 (25)		
	Very much	31 (29)	2 (6)	9 (12)		
	**Mean ± SD**	**1.77 ± 0.99**	**1.09 ± 0.92**	**1.23 ± 1.9**	**19.43**	**0.004**
11. You were concerned about the number of staff availability to take care of the COVID-19 patients[c]	Not at all	6 (6)	4 (12)	13 (18)		
	Slight	21 (19)	12 (36)	23 (32)		
	Moderate	45 (42)	13 (39)	25 (34)		
	Very much	36 (33)	4 (12)	12 (16)		
	**Mean ± SD**	**2.03 ± 0.87**	**1.52 ± 0.87**	**1.49 ± 0.97**	**18.02**	**0.006**
12. You were concerned about the availability of hospital beds/isolation units[c]	Not at all	5 (5)	3 (9)	11 (15)		
	Slight	23 (21)	16 (49)	23 (32)		
	Moderate	37 (34)	7 (21)	25 (34)		
	Very much	43 (40)	7 (21)	14 (19)		
	**Mean ± SD**	**2.09 ± 0.89**	**1.55 ± 0.938**	**1.58 ± 0.97**	**20.70**	**0.002**
13. You were concerned about the adequate supply of cleaning and sanitation material[c]	Not at all	5 (5)	6 (18)	18 (25)		
	Slight	27 (25)	16 (49)	20 (28)		
	Moderate	37 (34)	9 (27)	17 (24)		
	Very much	39 (36)	2 (6)	17 (24)		
	**Mean ± SD**	**2.02 ± 0.89**	**1.21 ± 0.82**	**1.46 ± 1.11**	**29.23**	**< 0.001**
14.You were concerned about the fast changing guidelines for managing patients[c]	Not at all	6 (6)	5 (15)	16 (22)		
	Slight	27 (25)	14 (42)	26 (36)		
	Moderate	43 (40)	9 (27)	17 (23)		
	Very much	32 (30)	5 (15)	14 (19)		
	**Mean ± SD**	**1.94 ± 0.88**	**1.42 ± 0.94**	**1.40 ± 1.0**	**19.77**	**0.003**
15. You were concerned about the level of training of Health Care Workers[c]	Not at all	10 (9)	3 (9)	21 (29_		
	Slight	27 (25)	13 (39)	16 (22)		
	Moderate	40 (37)	9 (27)	18 (25)		
	Very much	31 (29)	8 (24)	18 (25)		
	**Mean ± SD**	**1.85 ± 0.945**	**1.67 ± 0.96**	**1.45 ± 1.2**	**17.02**	**0.009**
16. You were concerned about reponse of the public to the lockdown[a]	Not at all	6 (6)	2 (6)	12 (17)		
	Slight	21 (19)	6 (18)	24 (34)		
	Moderate	32 (30)	14 (42)	17 (24)		
	Very much	49 (45)	11 (33)	18 (25)		
	**Mean ± SD**	**2.15 ± 0.92**	**2.03 ± 0.88**	**1.58 ± 1.05**	**17.93**	**0.006**
17. You were concerned if you test positive, because you don't have self-quarantine space at home[b]	Not at all	28 (26)	22 (67)	39 (53)		
	Slight	20 (19)	5 (15)	11 (15)		
	Moderate	21 (19)	0	9 (12)		
	Very much	39 (36)	6 (18)	14 (19)		
	**Mean ± SD**	**1.66 ± 1.2**	**0.70 ± 1.12**	0.97 ± 1.2	**27.01**	**< 0.001**
18. You were concerned if test positive, because you might infect family members unintentionally[b]	Not at all	10 (9)	4 (13)	8 (11)		
	Slight	9 (8)	6 (19)	13 (18)		
	Moderate	22 (20)	7 (21)	19 (26)		
	Very much	67 (62)	15 (47)	33 (41)		
	**Mean ± SD**	**2.35 ± 3.0**	**2.03 ± 1.09**	**2.35 ± 3.0**	**7.43**	**0.283**

19. You were concerned if you test positive, because I might be isolated without contact with family members[a]	Not at all	18 (17)	11 (33)	27 (37)		
	Slight	19 (18)	11 (33)	17 (23)		
	Moderate	27 (25)	5 (15)	14 (19)		
	Very much	44 (41)	6 (18)	15 (21)		
	**Mean ± SD**	**1.90 ± 1.12**	**1.18 ± 1.103**	**1.23 ± 1/.16**	**19.75**	**< 0.003**
20. You were concerned if you test positive, because of being stigmatized[b]	Not at all	28 (26)	21 (66)	40 (55)		
	Slight	23 (21)	6 (19)	18 (25)		
	Moderate	25 (23)	2 (6)	5 (7)		
	Very much	32 (30)	3 (9)	10 (14)		
	**Mean ± SD**	**1.56 ± 1/17**	**0.59 ± 0.98**	**0.79 ± 1.07**	**31.55**	**< 0.001**
21.You were concerned if you test positive, because of fear to die due to lack if specific treatment[b]	Not at all	22 (20)	14 (42)	31 (43)		
	Slight	24 (22)	9 (27)	23 (32)		
	Moderate	27 (250	7 (21)	8 (11)		
	Very much	35 (32)	3 (9)	11 (15)		
	**Mean ± SD**	**1.69 ± 1.13**	**0.97 ± 1.02**	**0.99 ± 1.07**	**23.18**	**0.001**
22. You were concerned to lose your job[a]	Not at all	24(22)	20 (61)	34 (47)		
	Slight	25 (23)	8 (24)	17 (23)		
	Moderate	25 (23)	3 (9)	9 (12)		
	Very much	33 (31)	2 (6)	13 (18)		
	**Mean ± SD**	**1.63 ± 1.14**	**0.61 ± 0.89**	**1.01 ± 1.2**	**25.61**	**< 0.001**

Correstponds to questions 8-12 on the original questionnaire. The questions were scored from 0 = no concern to 3 = concern. P value ≤ 0.05 was considered statistically significant. *Footnotes:* [a] *Not present in the analyzed figures.* [b] *Staff feeling during COVID-19 outbreak concerning themselves, family & financial stability (Fig 2).* [c] *Overall feeling of HOH staff concerning hospital safety (Fig 3)*
